# Comprehensive Analysis of VCAN Expression Profiles and Prognostic Values in HCC

**DOI:** 10.3389/fgene.2022.900306

**Published:** 2022-06-24

**Authors:** Guangshun Sun, Wubin Zheng, Pengyu Tan, Jin Zhou, Weiwei Tang, Hongyong Cao, Li Liu, Xuesong Shi, Zhouxiao Li, Wenling Zhang

**Affiliations:** ^1^ Department of General Surgery, Nanjing First Hospital, The Affiliated Nanjing Hospital of Nanjing Medical University, Nanjing, China; ^2^ Department of Food Science and Engineering, Nanjing Xiaozhuang University, Nanjing, China; ^3^ Hepatobiliary/Liver Transplantation Center, The First Affiliated Hospital of Nanjing Medical University, Key Laboratory of Living Donor Transplantation, Chinese Academy of Medical Sciences, Nanjing, China; ^4^ First Teaching Hospital of Tianjin University of Traditional Chinese Medicine, National Clinical Research Center for Chinese Medicine Acupuncture and Moxibustion, Tianjin, China; ^5^ Department of Plastic and Reconstructive Surgery, Shanghai Ninth People’s Hospital, Shanghai Jiao Tong University School of Medicine, Shanghai, China; ^6^ Department of Gastroenterology, The First Affiliated Hospital of Nanjing Medical University, Nanjing, China

**Keywords:** VCAN, hepatocellular carcinoma, prognosis, invasion, immune

## Abstract

**Background:** Hepatocellular carcinoma (HCC) is the world’s most common cause of cancer death. Therefore, more molecular mechanisms need to be clarified to meet the urgent need to develop new detection and treatment strategies.

**Methods:** We used TCGAportal, Kaplan–Meier Plotter, the Cistrome DB Toolkit Database, MExpress, GEPIA2, and other databases to discuss the expression profiles, possible biological function, and potential prognostic value of versican (VCAN) in HCC. We conducted cell experiments such as Transwell migration and invasion assays, wound healing assay, and CCK8 experiment to explore the function of VCAN in HCC.

**Result:** We selected three HCC transcriptome databases GSE124535, GSE136247, and GSE144269 and analyzed the overexpressed genes contained in them. The overlapping genes were found by the Venn map, and two interacting network modules were found by Mcode. Module 1 was mainly related to mitosis and cell cycle, and module 2 was mainly related to EMT, angiogenesis, glycolysis, and so on. We found that the seed gene in module 2 is VCAN. Data from TCGAportal showed that compared with normal tissues, the expression of VCAN was up-regulated in HCC tissues. The patients with high expression of VCAN had shorter distant recurrence-free survival and overall survival. Multiple possible VCAN interactions had also been identified. These results revealed that the level of VCAN was higher in the subtypes of HCC with higher malignant degree and was connected to the poor prognosis. In addition, the treatment of VCAN with DNA methyltransferase inhibitors and transcription factor inhibitors may improve the prognosis of patients with HCC.

**Conclusion:** Our findings systematically elucidated the expression profile and different prognostic values of VCAN in HCC, which may provide new therapeutic targets and potential prognostic biomarkers for HCC patients.

## Introduction

HCC is the world’s most common cause of cancer death, the fifth most common cancer in the United States, and the only cancer with an increasing annual incidence among the top five fatal cancers (Siegel et al., 2019). The prognosis of HCC is extremely poor, with a 5-year average survival rate of less than 10% (Bray et al., 2018). In addition, only 5%–15% of patients with HCC are eligible for surgical resection at the early stage, while most patients with HCC are diagnosed with advanced cancer. Advanced treatment includes transarterial chemoembolization (TACE) and oral sorafenib chemotherapy. However, less than 1/3 of patients benefited from treatment, and drug resistance was evident within 6 months after the start of treatment (El-Serag et al., 2008). In addition, immune checkpoint inhibitors (ICIs) have gradually become a hot field of cancer treatment. In some economically developed countries, more than half of metastatic cancer patients are eligible for ICI treatment. As of December 2021, there are eight ICI-related drugs approved, and they are used to treat up to 17 different malignancies (Haslam and Prasad, 2019). At the same time, these drugs are increasingly used in multiple (neo)adjuvant and maintenance treatments, and ICIs are frequently used in combination regimens (Vilgelm et al., 2016). Immune checkpoints are receptors expressed by immune cells that dynamically regulate immune homeostasis and are particularly relevant to T-cell function (Blank et al., 2019). The PD1/PDL1 monoclonal antibody, which has recently attracted much attention, is one of them. However, recent basic and clinical studies have shown that the cancellation of immune checkpoints will bring many unavoidable side effects and even endanger the lives of patients. At the same time, drug resistance is also a problem that cannot be ignored (Sun et al., 2022). Therefore, exploring biomarkers with high specificity and sensitivity or looking for new molecular targets can not only help clinicians to predict the prognosis of patients but also clarify the potential mechanism of HCC, which has long-term significance.

VCAN is a chondroitin sulfate proteoglycan, a major component of extracellular matrix (ECM), which provides hydration and lose matrix in disease progression and critical events (Kinsella et al., 2004; Wu et al., 2005). VCAN refers to a complex molecule that covers the glycosaminoglycan side chain and modular core protein domain and has a series of synthetic procedures and processes to regulate these elements (Wight, 2002). VCAN can affect the process of cell adhesion, proliferation, migration, and angiogenesis, which seriously affects the morphogenesis and maintenance of tissue (Rahmani et al., 2006).In addition, VCAN involves many pathological steps, including axonal outcome, central nervous mechanism injury, hair follicle circulation, tendon remodeling, and atherosclerotic vascular disease (Du et al., 2013; Shen et al., 2020). However, the detailed function and molecular mechanism of VCAN in HCC are still unclear. Therefore, in this study, we studied the expression, molecular mechanism, and clinical correlation of VCAN in HCC.

## Result

### VCAN Might Be Related to EMT, Angiogenesis, and Glycolysis

**FIGURE 1:**
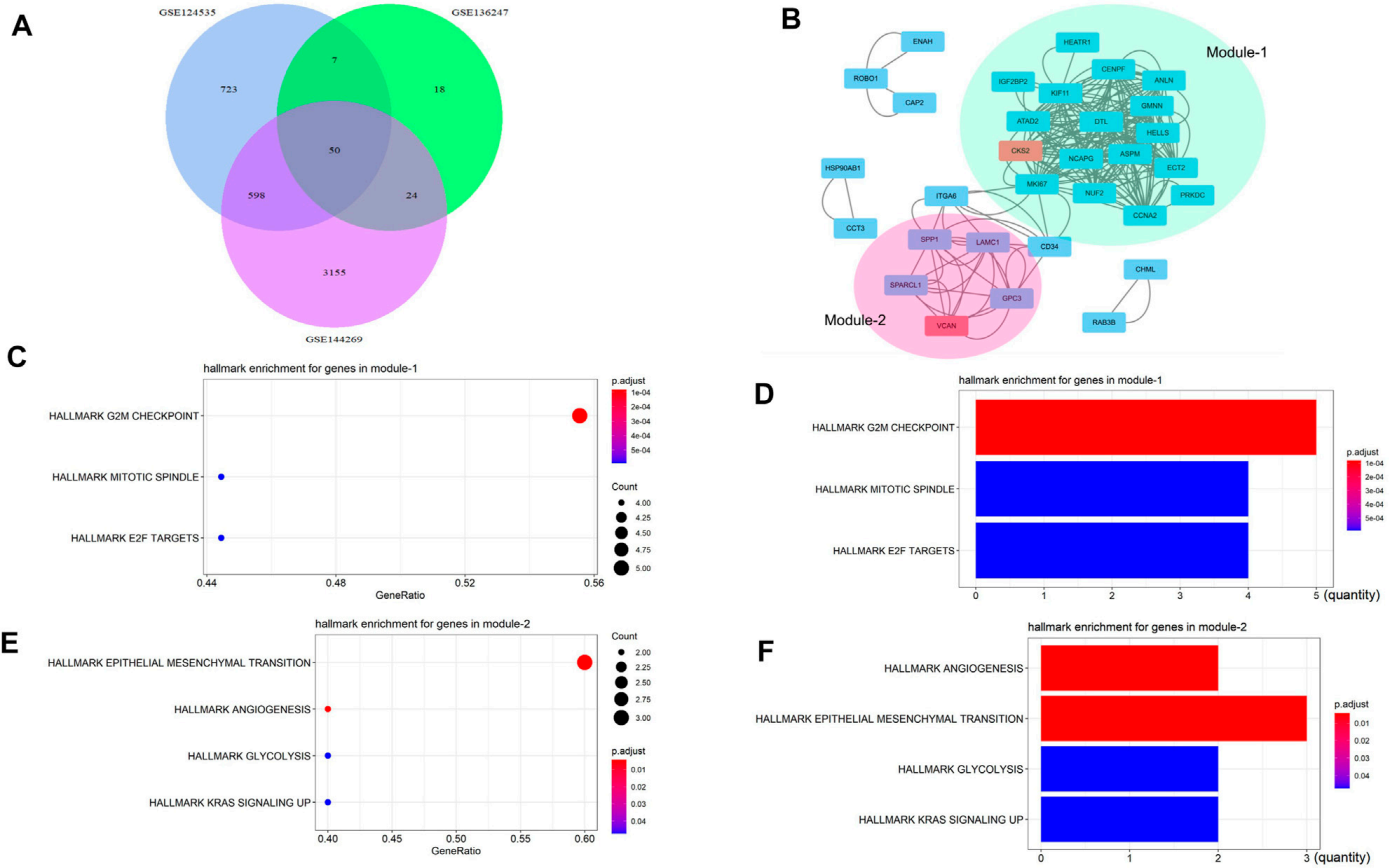
VCAN might be related to EMT, angiogenesis, and glycolysis. **(A)** We selected three liver cancer transcriptome databases GSE124535, GSE136247, and GSE144269, and used GEO2R to analyze the overexpressed genes contained in them. The overlapping genes were found by the Venn map. **(B)** Two interacting networks module were found by Mcode. **(C–D)** Module1 is mainly related to mitosis and the cell cycle. **(E–F)** Module2 is mainly related to EMT, angiogenesis, glycolysis, and so on.

We selected three HCC transcriptome databases GSE124535, GSE136247, and GSE144269, and used GEO2R to analyze the overexpressed genes contained in them. The overlapping genes were found by the Venn map (Figure 1A), and two interacting networks module, were found by Mcode (Figure 1B). Module1 was mainly related to mitosis and cell cycle (Figure 1C,D), and module2 was mainly related to EMT, angiogenesis, glycolysis, and so on (Figure 1E,F). We found that the seed gene in module2 was VCAN.

### VCAN Was Over-Expressed in HCC

**FIGURE 2:**
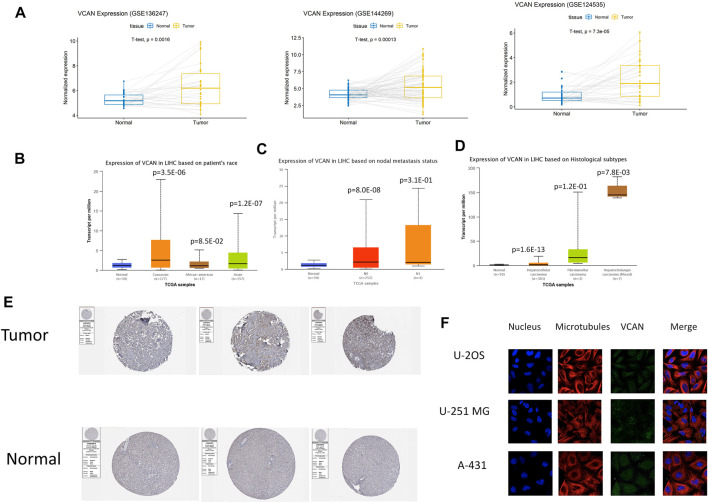
VCAN expression overview. **(A)** The expression level of VCAN mRNA in HCC is obviously more than that in normal tissues. **(B–D)** Discrepancy in VCAN mRNA expression is hinged on race, nodal metastasis status, and histological subtypes. **(E)** The expression level of VCAN in normal tissue and HCC tissue. **(F)** VCAN is in the same position as microtubule proteins in the cytoplasm of U-2 OS, A-431, and U-251 MG cells.

The three databases GSE124535, GSE136247, and GSE144269 all showed that the expression of VCAN in HCC tissue is higher than in normal tissue (Figure 2A), Next, we used UALCAN to conduct a more comprehensive analysis of VCAN mRNA expression in HCC. Subgroup analysis based on race, nodal metastasis status, and histological subtypes showed significantly higher VCAN mRNA levels in HCC patients than in healthy individuals (Figure 2B–D). The results identified, to a certain extent, the population that could benefit from VCAN-targeted liver cancer treatment in the future. The analysis of the Human Protein Atlas data indicated that VCAN staining was stronger in HCC tissue than in normal liver tissue (Figure 2E). RNA expression and protein localization results for VCAN based on data generated in the Human Protein Atlas project are VCAN detected in the vesicles, and expected to be secreted. When VCAN is secreted outside the cell, it is mainly present in the ECM. The subcellular location of VCAN was further confirmed based on immunofluorescence analysis of all study cell lines and all antibodies tested (Figure 2F).

### Research Results of VCAN in the Single-Cell Level

**FIGURE 3:**
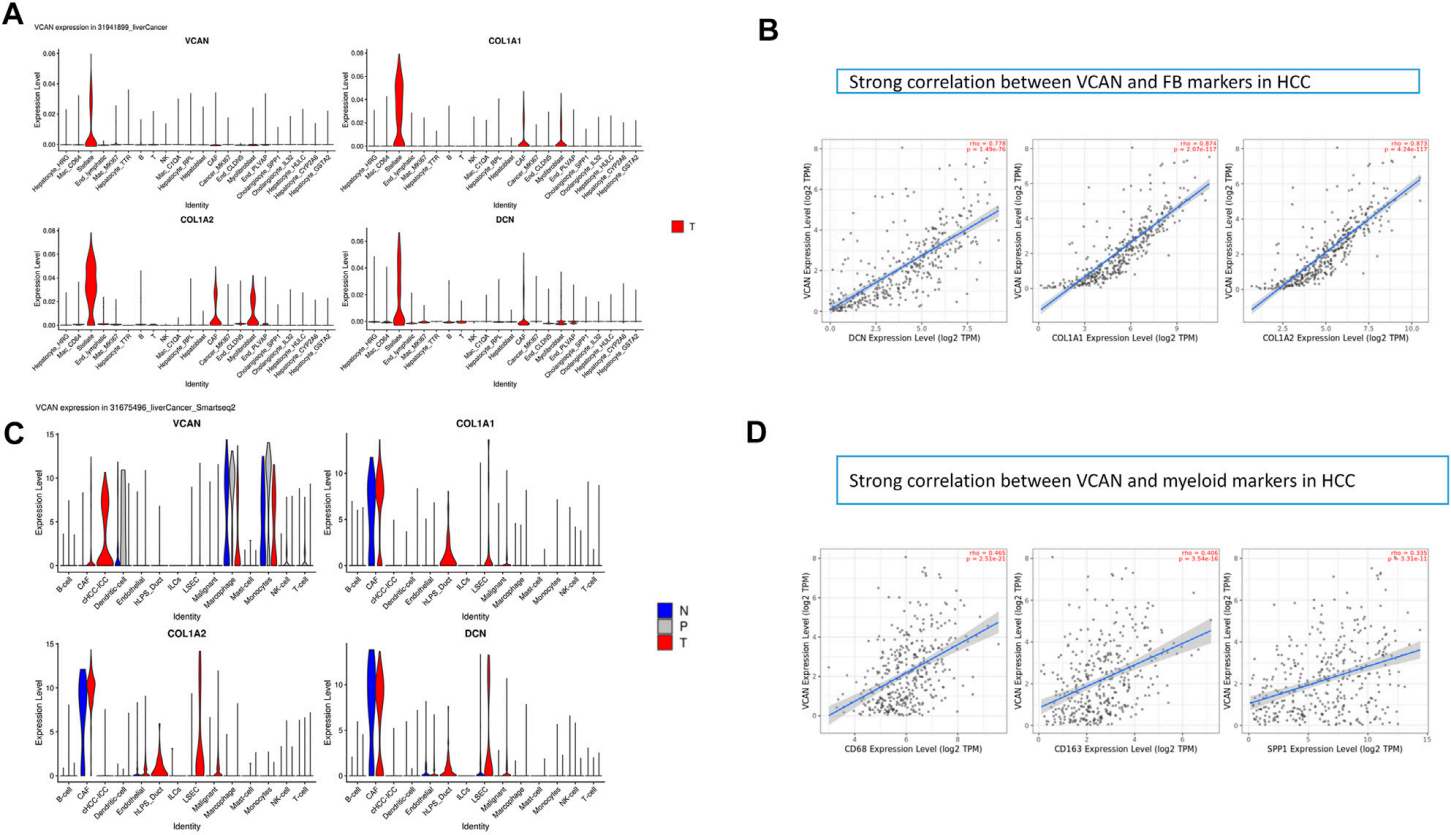
Research results of VCAN in a single cell. **(A)** VCAN is expressed in fibroblasts and has a similar expression pattern to classical fibroblast markers such as COLA1/2, DCN. **(B)** There is a strong correlation between VCAN and FB markers in HCC. **(C)** VCAN is highly expressed in the myeloid. **(D)**Some correlation between VCAN and myeloid markers in HCC.

We studied the expression of VCAN at the single-cell level. Stellate is a subtype of fibroblasts. Studies have shown that VCAN was expressed in fibroblasts and had a similar expression pattern to classical fibroblast markers such as COLA1/2 and DCN (Figure 3A). Furthermore, data indicated that there was a strong correlation between VCAN and fibroblast markers in HCC (Figure 3B). In addition to fibroblasts, VCAN was also highly expressed in myeloid (Figure 3C). Some correlation between VCAN and myeloid markers in HCC was exhibited in Figure 3D.

### VCAN Expression Was Strongly Associated With Clinical Outcome

**FIGURE 4:**
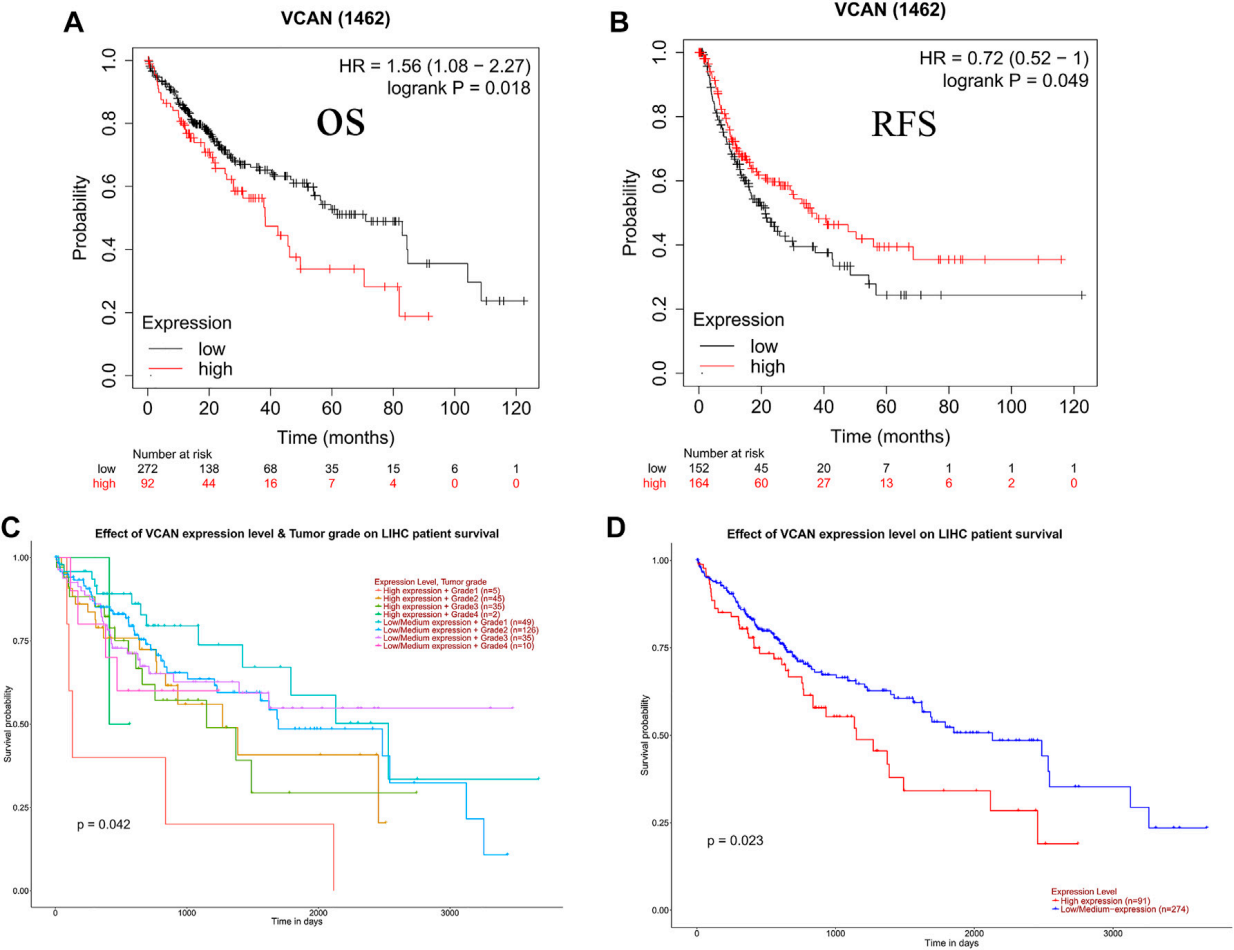
Clinical role of VCAN in HCC. **(A-D)** The patient has a poor OS and poor RFS with a high level of VCAN.

The prognostic potential of VCAN in HCC was further examined using Kaplan–Meier Plotter. Results indicated that the overall survival of the population with low VCAN expression was significantly higher than that of the population with high VCAN expression (Figure 4A,D). But surprisingly, the recurrence-free survival of the low VCAN-expressing population appears to be lower than the high VCAN-expressing population, but with a *p* value of 0.049 (Figure 4B). In Grade 2 and Grade 3 HCC patients, the survival of patients with low VCAN expression was significantly higher than that of HCC patients with high VCAN expression. Due to the small number of patients in Grade 1 and Grade 4 counted, the differences between these two populations cannot be accurately counted (Figure 4C).

### VCAN Played a Promoting Role in HCC Cells *In Vitro*

**FIGURE 5:**
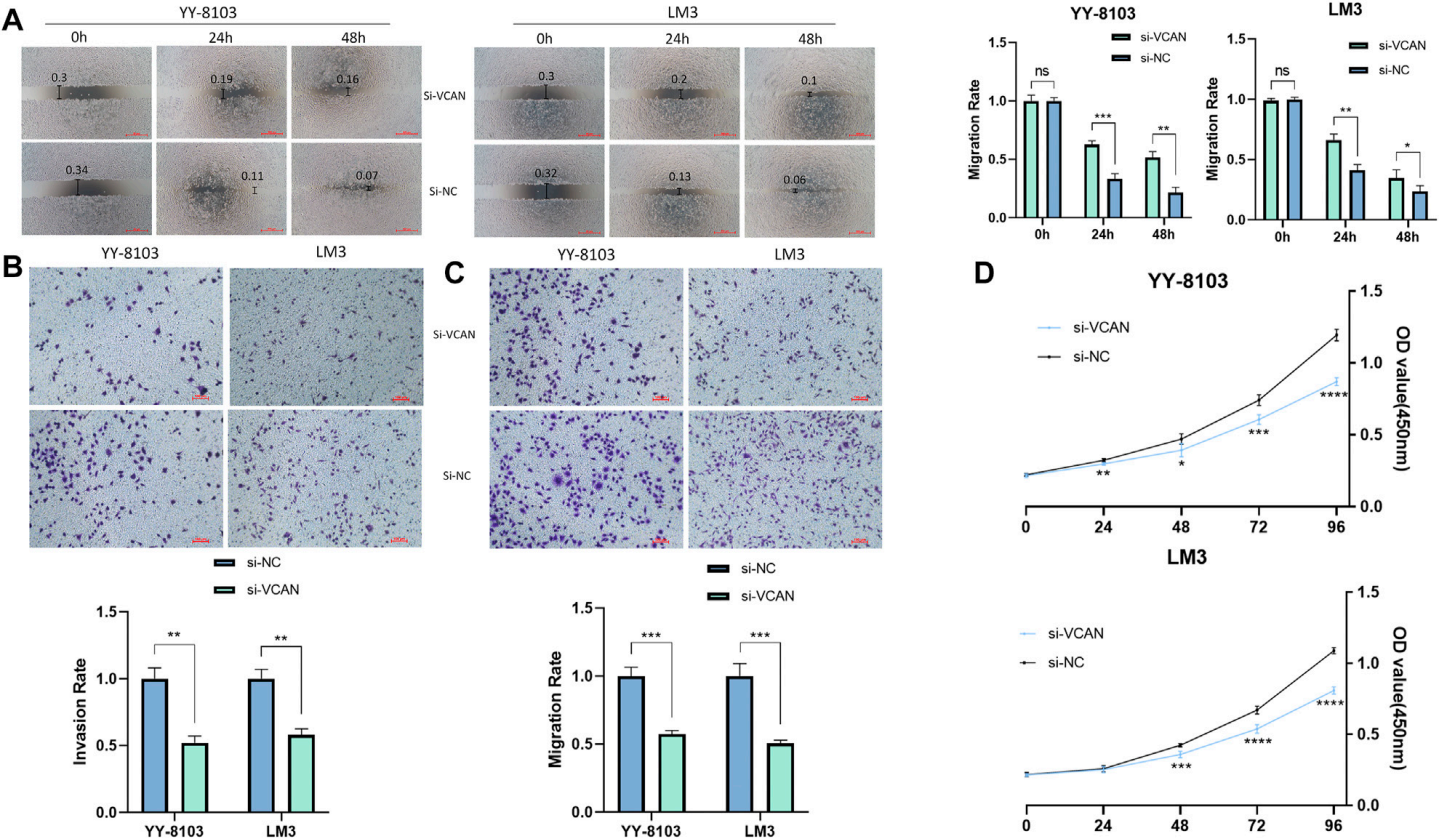
Artificial reduction of VCAN expression can effectively inhibit the proliferation of HCC cells. **(A)** Scratch assay was used to reduce the expression of the VCAN gene. **(B)** When VCAN expression was inhibited, the migration of HCC cells was also inhibited. **(C)** By knocking down the expression of VCAN, the invasion of HCC was effectively prevented. **(D)** CCK8 assay confirmed that the inhibition of VCAN by si-VCAN could slow down the proliferation of HCC cells. *p < 0.05; **p < 0.01; ***p < 0.001; ****p < 0.0001.

**FIGURE 6:**
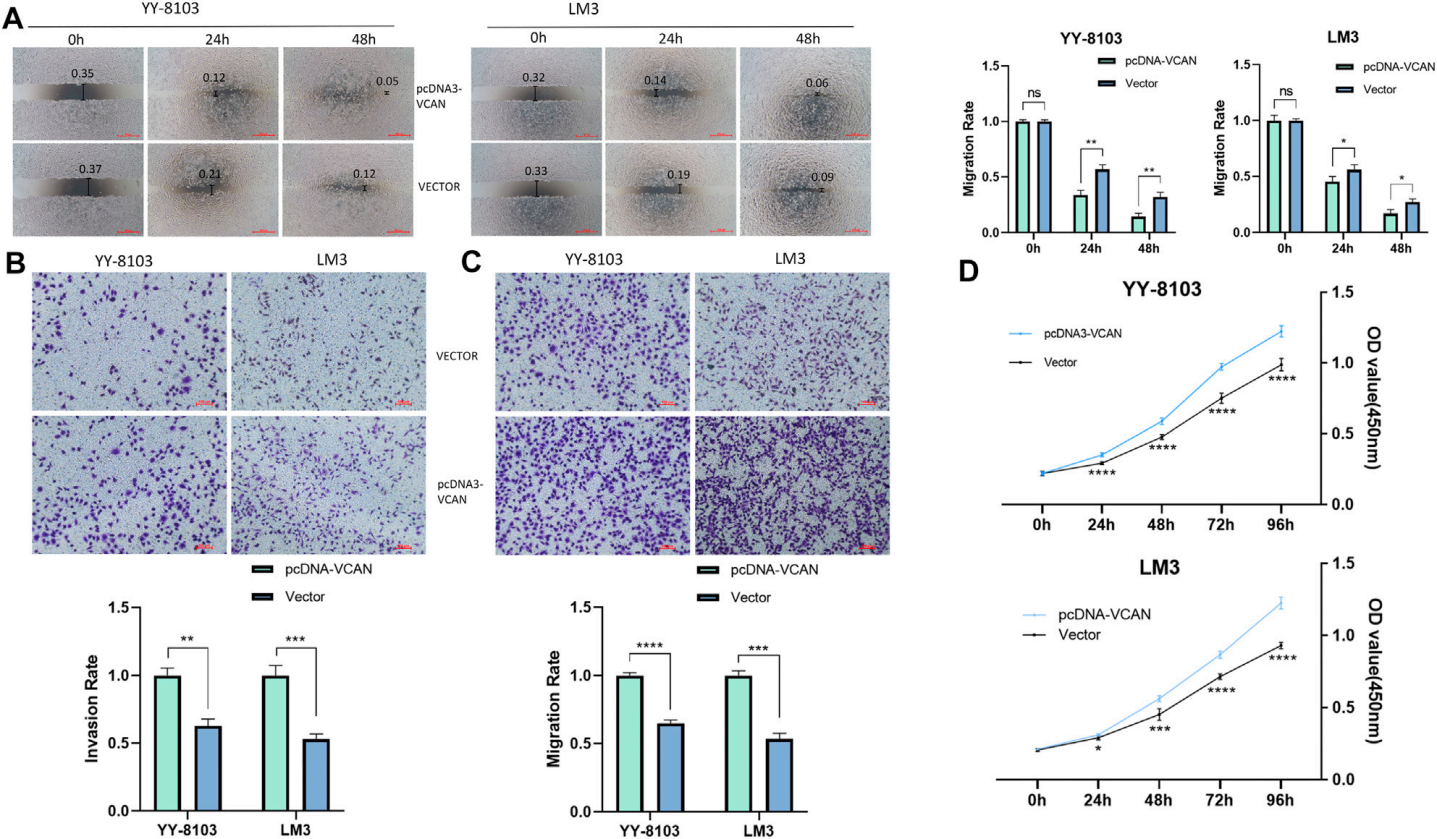
Artificial up-regulation of VCAN expression can effectively accelerate the proliferation of HCC cells. **(A)** Scratch assay was used to increase the expression of VCAN gene. **(B)** When VCAN expression was up-regulated, the migration of HCC cells was also promoted. **(C)** By up-regulating the expression of VCAN, the invasion of HCC was effectively promoted. **(D)** CCK8 assay confirmed that the overexpression of VCAN could accelerate the proliferation of HCC cells.*p < 0.05; **p < 0.01; ***p < 0.001; ****p < 0.0001.

The results of the scratch assay showed that in the HCC cell line, the scratch closure rate of inhibiting VCAN was significantly lower than that of the control group (Figure 5A). Compared with the control group in the confluence monolayer transwell experiment of cultured HCC cell line, si-VCAN inhibited the relative migration and invasion rate of VCAN (Figure 5B,C). Plate cloning and CCK-8 assay showed that VCAN gene knockout significantly inhibited the proliferation of YY-8103 and LM3 cells compared with the control group (Figure 5D). The overexpression of VCAN has the opposite effect (Figure 6A–D). These results suggested that inhibition of VCAN could delay the proliferation, invasion, and migration of HCC *in vitro*.

### The Transcription Factors (TFs) That May Affect the Transcription of the VCAN Gene

**FIGURE 7:**
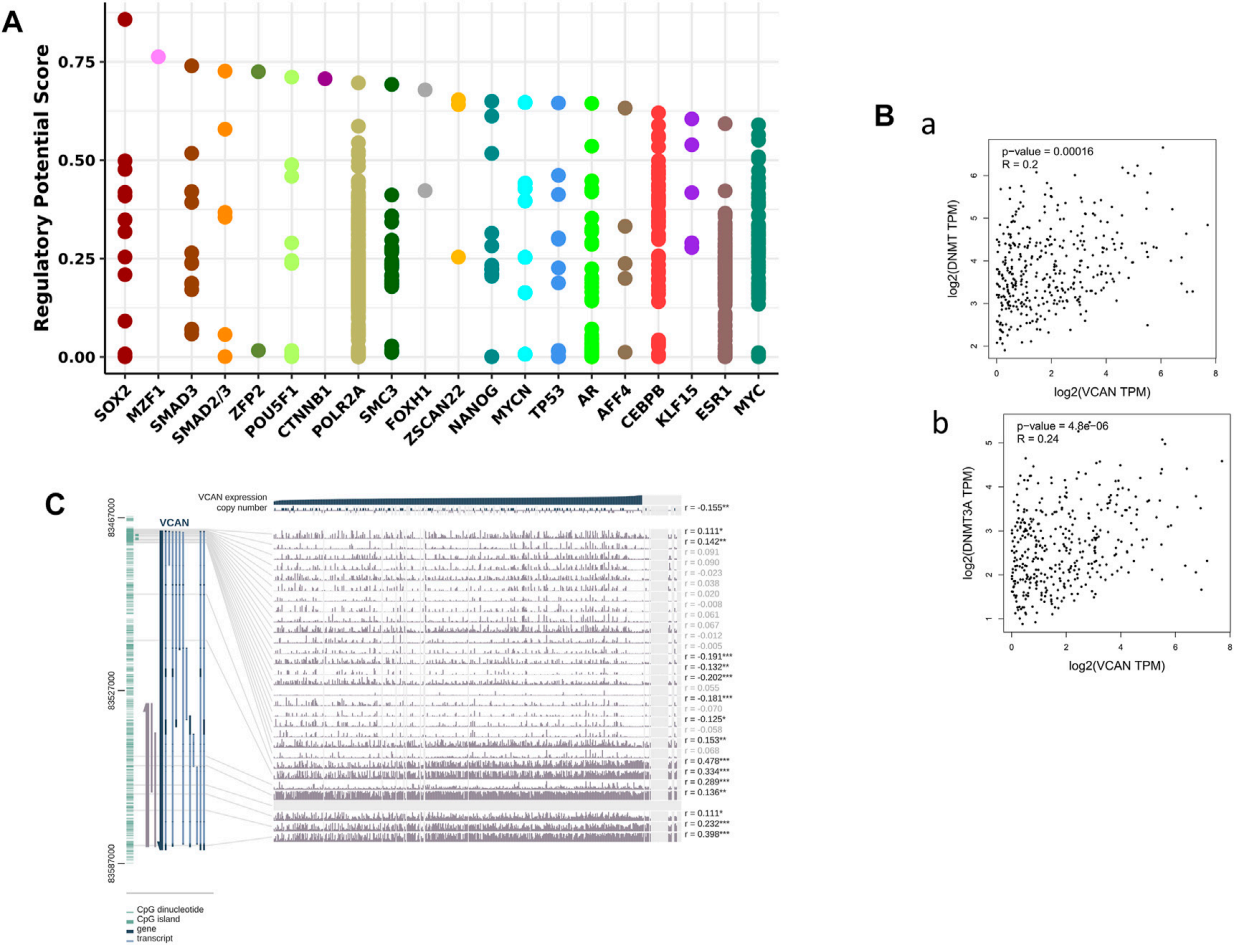
VCAN may had the ability to control HCC-related genes and TFs in HCC. **(A)** VCAN was regulated by those 20 most likely TFs in different human cancers. **(B)** There’s a connection between VCAN mRNA expression and DNA methyltransferase (DNMT) expression. **(C)** VCAN DNA methylation modification in HCC.

In order to identify the members of the molecular network that may regulate the expression of VCAN, we detected the TF that may affect the transcription of the VCAN gene. First, the 20 most regulated TF in human cancers were identified using Cistrome DB Toolkit (Figure 7A). We reviewed the relevant literature and found that transcription factors such as SOX2, SMAD3, CTNNB1, and TP53 have been reported to play an important role in liver cancer. Previous studies have shown that the high expression of SOX2 is associated with metastasis and a low survival rate of HCC. Hepatoma cells overexpressing SOX2 are characterized by active epithelial-mesenchymal transition, showing a stronger ability for transpose invasion, soft agar colonization, and spheroid formation (Sun et al., 2013). Hepatoma cells release exosomes containing SMAD family member 3 (SMAD3) protein and mRNA and transfer them to isolated hepatoma cells to promote their adhesion. These exosomes can induce the enhancement of SMAD3 signal transduction and adhesion ability of the recipient hepatoma cells. In addition, the research also found that there are abundant SMAD3 exosomes in the peripheral blood of patients with HCC, and its level is related to the stage of the disease and the expression of Smad3 in the primary tumor (Fu et al., 2018). In addition to these, Wnt/CTNNB1 mutation is a characteristic of immune rejection (cold tumor) and maybe a biomarker for predicting drug resistance of immunosuppressants at immune checkpoints in HCC (Pinyol et al., 2019).

### VCAN mRNA Expression Is Positively Correlated With DNMT

Previous studies have shown that DNA methylation plays an important role in HCC (Xu et al., 2017). In addition, the methylation of CpG island in the promoter region of the gene prevents some TF from binding to DNA, thus inhibiting gene transcription. Therefore, we used MEXPRESS to examine the DNA methylation modification of the VCAN gene in HCC (Figure 7C). Interestingly, in the GEPIA 2 database, there was also a positive correlation between the expression of VCAN and DNA methyltransferase (DNMT) in HCC (Figure 7B). These results suggest that transcription factors and DNA methylation may play an important role in the process of HCC by regulating the expression of VCAN.

### miRNA, circRNA, and RBP Interact With VCAN in HCC

**FIGURE 8:**
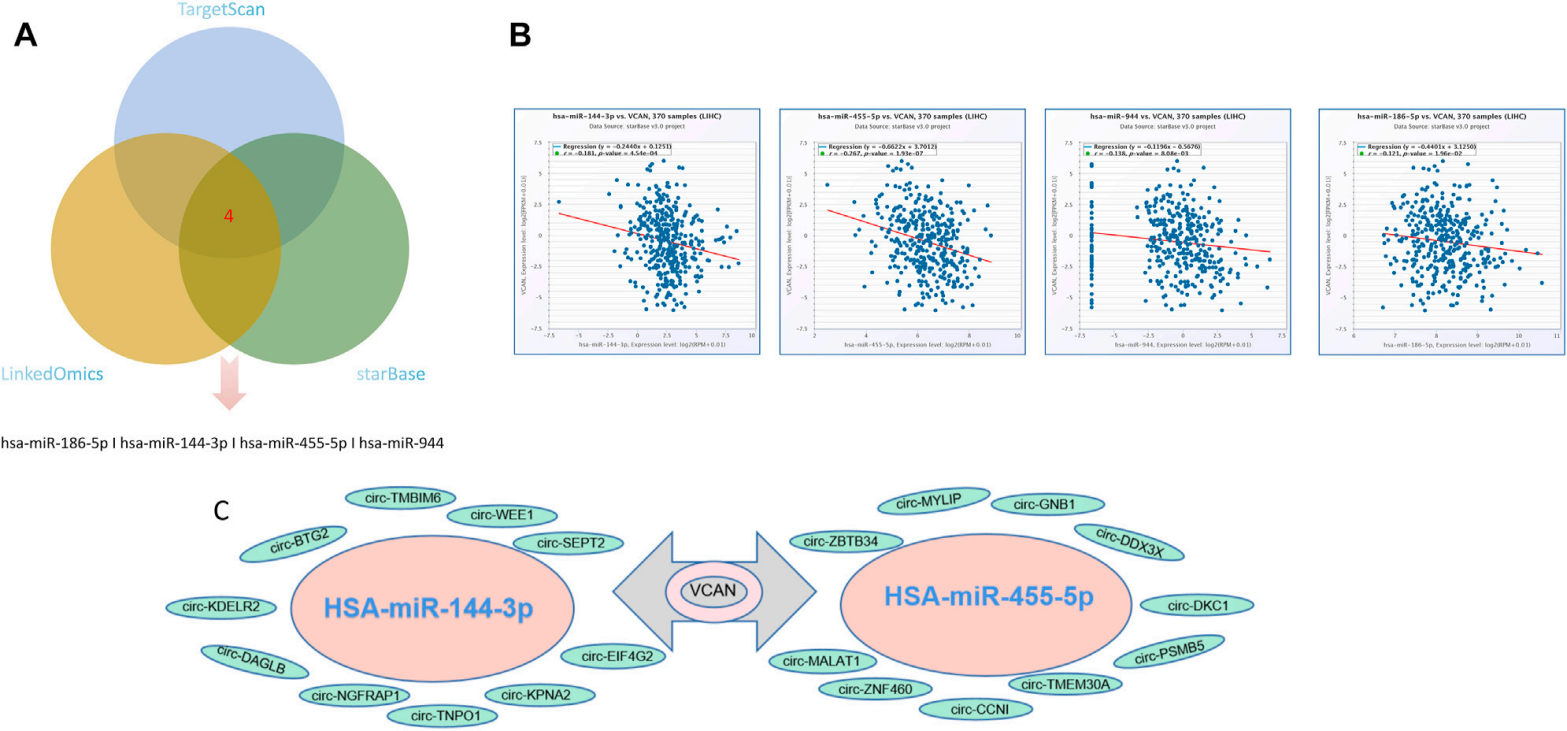
CircRNAs and miRNAs that might regulate VCAN. **(A)** From three miRNA prediction datasets, we select four miRNAs that might regulate VCAN. **(B)** The four miRNAs are negatively correlated with VCAN mRNA expression. **(C)** Top 10 circRNAs interacting with HSA-miR-144-3p or HSA-miR-455-5p identified by starBase.

By mining the three databases of LinkedOmics, Starbase, and Target can, it was found that four common miRNA were down-regulated in HCC: hsa-miR-144-3p, hsa-miR-455-5p, hsa-miR-944, and hsa-miR-186-5p (Figure 8A). Previous studies have shown that the expression of hsa-miR-144-3p in HCC is significantly higher than that in adjacent tissues, and the ratio of HSA144-3p/hsA-miR-21-5p increases significantly during the occurrence of HCC, which is even better than alpha-fetoprotein in ROC curve analysis, suggesting that HSA144-3p may be an excellent predictive marker of liver cancer (Pu et al., 2018). In addition, HSA-miR-455-5p has also been proved to be involved in the occurrence and development of liver cancer (Wang et al., 2021). Furthermore, the analysis of Starbase showed that the expression of the four miRNAs was negatively correlated with the expression of VCAN in HCC (Figure 8B). Since circRNA can further regulate gene expression through sponge miRNAs, 10 circRNAs of sponge hsa-miR-455-5p, and hsa-miR-144-3p are also identified in HCC (Figure 8C). RNA binding proteins (RBP) are important post-transcriptional regulators, and different RBPs can interact with many RNA binding domains. The development of cancer is often accompanied by abnormal interactions between RBPs and RNA (Deng et al., 2018). We used Starbase to mine 20 RBP most likely to interact with VCAN mRNA in hepatoma cell line HepG2.

### VCAN Expression Was Correlated With Immune Factors

**FIGURE 9:**
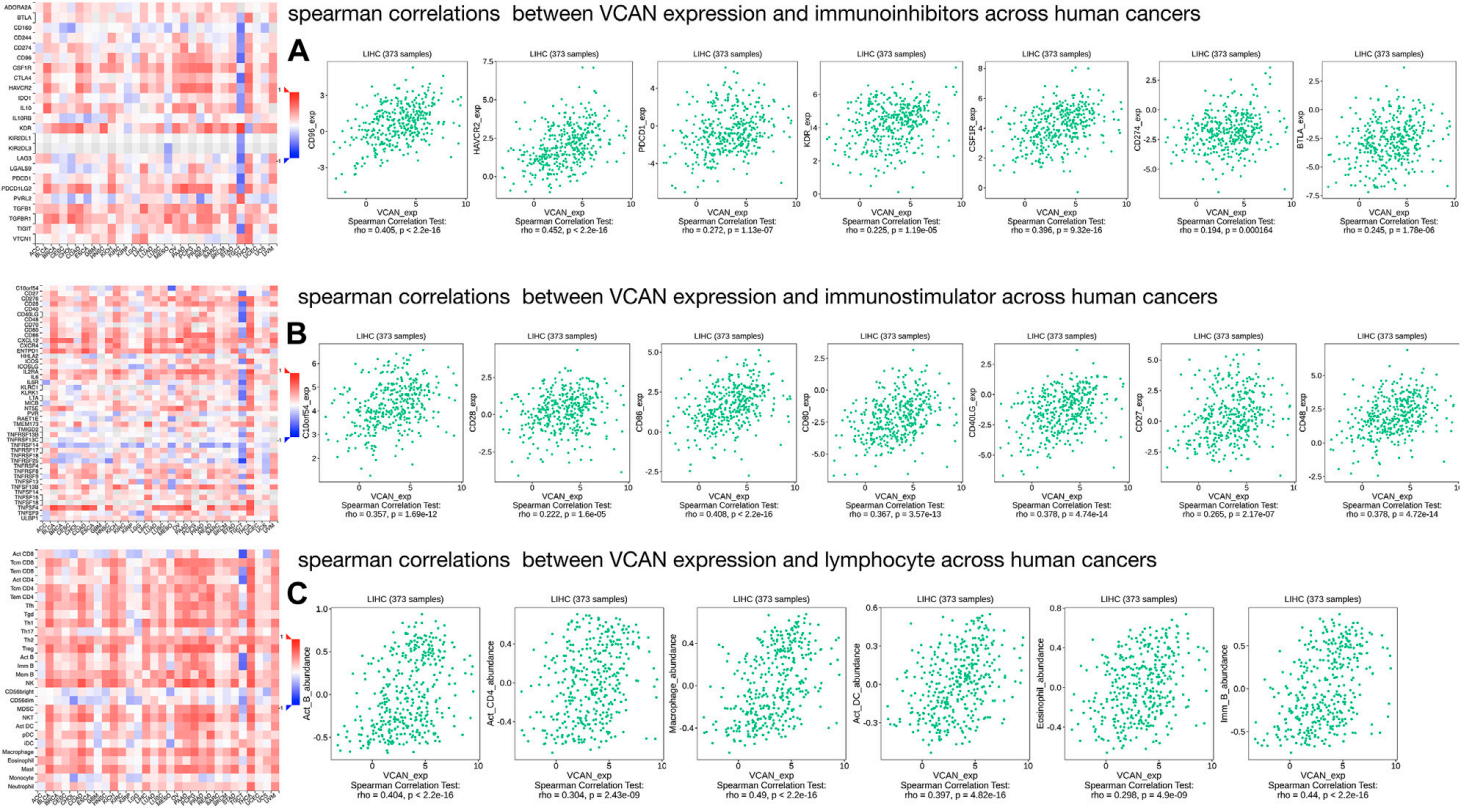
Three types of cancer-related immune factors are related to VCAN. **(A)** Correlation between VCAN expression and immunoinhibitors in HCC. **(B)** Correlation between VCAN expression and immunostimulator in HCC. **(C)** Correlation between VCAN expression and lymphocyte in HCC.

Existing studies have confirmed that the immune system is closely related to the occurrence and development of tumors. Therefore, we studied the relationship between the expression of VCAN and immune factors. Figure 9A–C showed that there was a strong correlation between the expression of immune inhibitors, immunostimulators, and lymphocytes and the expression of VCAN.

## Materials and Methods

### VCAN Expression Level Analysis

TCGAportal (www.tcgaportal.org) was used to study the expression of VCAN in different tumor tissues and corresponding paracancerous tissues. The human protein map (https://www.proteinatlas.org/) database contains pathological and genetic information from many reports from a variety of tissues and cells. We used it to detect the expression of VCAN in different tissues and the localization of VCAN mRNA in cells. Next, we used UALCAN (http://ualcan.path.uab.edu/) to compare the expression of VCAN in patients with HCC of different races, ages, and histological subtypes. Finally, the significance of the observed difference was evaluated by the Wilcoxon rank-sum test.

### Relapse and Survival Analysis

Kaplan–Meier Plotter (http://kmplot.com/analysis/index.php?p=background) is a free online database, built by using gene expression data and survival data from a variety of cancer patients including HCC (Gyorffy et al., 2010; Gyorffy et al., 2013; Szasz et al., 2016). We used this online database to explore the relationship between the expression of VCAN and OS and RFS of patients with HCC. Kaplan–Meier survival plots were used to compare OS and RFS in HCC patients with high VCAN expression and those with low VCAN expression, and 95% confidence interval hazard ratios and log-rank *p* values were calculated.

### TF Identification

The Cistrome DB Toolkit database (http://dbtoolkit.cistrome.org) is a resource of human and mouse cis-regulatory information, including about 47,000 human and mouse samples with about 24,000 newly collected data sets compared with 2 years ago. Users can use this database to search for TFs related to the regulation of target genes in order to identify binding factors, histone modifications, and chromatin accessibility in a genomic interval of interest up to 2 Mb in length. Once users provide the overlap with the particular genomic interval sets, similar ATAC-seq, DNase-seq, and ChIP-seq samples can be determined (Mei et al., 2017; Zheng et al., 2019). We used the Cistrome Database Toolkit to search for TFs that were most likely to increase VCAN expression.

### DNA Methylation Modification Analysis

MEXPRESS (https://mexpress.be/), a user-friendly database tool for the visualization and interpretation of TCGA data, can be used to study TCGA expression, DNA methylation status, and clinical data and the relationships between them (Koch et al., 2015). In this research study, we use this database tool to study the methylation status of VCAN mRNA and the relationships between VCAN mRNA expression and different clinical characteristics in HCC patients.

### Gene Correlation Analysis

GEPIA2 (http://gepia2.cancer-pku.cn/#index), an open-access dataset, can be used to study RNA sequencing expression data from 9,736 tumors and 8,587 normal samples derived from the TCGA and GTEx projects. The dataset provides tumor/normal differential expression analysis, profiling according to cancer types or pathological stages, patient survival analysis, similar gene detection, correlation analysis, and dimensionality reduction analysis. In this study, we used GEPIA2 to synthetically analyze the correlations between all-important genes.

### Identification of miRNAs and circRNAs That Target VCAN

TargetScanHuman (http://www.target scan.org/vert_71/) has the ability to search for the presence of conserved 8mer, 7mer, and 6mer sites that match the seed region of each miRNA to predict biological targets of miRNAs. starBase v3.0 (http://starbase.sysu.edu.cn/index.php), an open-source platform for the identification of the interactions between miRNA to lncRNA, RBP to lncRNA, miRNA to mRNA, RNA to RNA, ncRNA to RNA, and RBP to mRNA from CLIP-seq, degradome-seq, and RNA-RNA interactome data. These two databases were used to confirm the potential miRNAs that bind to VCAN mRNA. In addition, starBase v3.0 was used to perform circRNA prediction, miRNA survival analysis, and analysis of correlations between miRNAs and VCAN mRNA.

### Protein–Protein Interaction and Functional Enrichment Analysis

Metascape (http://metascape.org/gp/index.html#/main/step1), a web portal, combines 40 independent knowledge bases’s functional enrichment, interactome analysis, gene annotation, and membership search. It promotes comparative analysis of multiple independent and orthogonal experiments across datasets (Zhou et al., 2019). STRING (https://string-db.org/cgi/input.pl) is a database that you can use to search for protein-protein interactions you are interested in, including direct (physical) and indirect (functional) connections; The conclusions obtained are comprehensively calculated and predicted, and knowledge transfer between organisms and interactions summarized in other (main) databases (Szklarczyk et al., 2019). We use STRING to create an interaction network between VCAN and other important proteins.

### Immune-Related Analysis

DISIDB (http://cis.hku.hk/TISIDB/index.php) is a web portal. Multiple heterogeneous data types were integrated to analyze the interaction between tumor and immune system in this web portal (Ru et al., 2019). We use it to analyze the spearman correlations between VCAN expression and immunostimulation, immune inhibitors, and lymphocytes across human cancers.

### Cell Culture and Transfection

We used RPMI 1640 medium, which contains 10% fetal bovine serum, to cultivate YY-8103 and LM3 cell lines in a 5% CO_2_ incubator with penicillin (100 IU/ml) and streptomycin (100 mg/ml). The small interfering RNA target to VCAN (si-VCAN) and untargeted control siRNA were produced by HongXin company in Nanjing, China. We used the solution manufacturing by Applied biological materials company (Canada) and the Opti-MEM (Gibco, United States) to transfer. The target sequence of si-VCAN we obtained was as follows: si-RNA: 5‘-GGA​UAG​GCC​UCA​AUG​ACA​ATT-3’.

### Cell Proliferation Experiments

In the CCK8 experiment, we firstly transfected the YY-8103 and LM3 cells line and incubated at 37 °C. Then put the CCK8 solution (Biosharp, China) into each hole and incubate for 2 hours. The absorbance was detected at 450 nm at 0, 24, 48, 72, and 96 h. We did all the experiments three times.

### Transwell Migration and Invasion Assays

In accordance with the manufacturer's instructions, we vaccinated the YY-8103 and LM3 cells line at the upper chamber, and the culture was performed on a 200 μL serum-free 1640 medium. The matrigel mix (BD Biosciences,United States) covers the transwell chamber (Corning, United States) so that the invasion test can be realized and the matrigel mix is not needed for the migration experiment. The HCC cell chemical inducers made by RPMI 1640 medium and 10% FBS were lured to the bottom of the chamber. Incubation for 24 h, we fixed the color of the upper chamber. Then crystal violet (Kaigen, China) was used for dyeing for 15 min. We photographed and counted the cells in three fields in order to implement visualization.

### Wound Healing Assay

After culture on a six-well plate, we transfected YY-8103 and LM3. The artificial linear wound in the monolayer fused cell was removed by the standard 20 μL pipetting devices. The free-floating cells and debris in the well bottom were removed slowly. Inject it into the medium and put the well in an incubator to incubate at 37°C. The width of scratch clearance was recorded by an inverted microscope and taking pictures at 0, 24, and 48 h. The difference between the width of the original wound and the width of the process of quantitative cell migration was done three times.

### Statistical Analysis

GraphPad Prism 8(United States) was used to analyze the data. The data had statistical significance when the *p* value was less than 0.05. An independent *t*-test was used to compare continuous information between the two groups. Corresponding significance level was shown in those figures.

## Discussion

Previous studies have shown that VCAN is an EMT-related gene, which plays a role in promoting, leukemia, breast cancer, non-small cell lung cancer, and other cancers. However, the research on VCAN in HCC is still relatively rare, so we used a variety of databases to explore the expression of VCAN in HCC. As expected, VCAN is highly expressed in HCC.

We use some databases to observe the co-localization of VCAN and tubulin in cells. Some previous studies have shown that tubulin plays an important role in the cell cycle and cell proliferation. Many anti-tumor drugs kill cancer cells by changing the microstructure of cancer cells (Tangutur et al., 2017; Kostrhunova et al., 2019). Therefore, in the development and progression of HCC, VCAN is likely to interact with the microtubule structure to promote cancer. The above reminds us that the focus of future research can be on the co-localization and interaction of VCAN and tumor tubulin.

*In vitro* experiments showed that VCAN gene knockout inhibited the proliferation, invasion, and migration of HCC cells, while overexpression of VCAN had the opposite effect. The theory of cancer stem cells (CSC) provides a new perspective on the mechanism of tumorigenesis and metastasis (Haraguchi et al., 2006). Some recent studies have pointed out that DNA methylation is a potential epigenetic mechanism to maintain CSC. In addition, studies have shown that DNMT plays a vital role in CSC, and knocking out DNMT can reduce and inhibit the occurrence of tumors by limiting and reducing the CSC pool (Yatabe et al., 2001; Morita et al., 2013; Pathania et al., 2015). In summary, targeting epigenetic modifiers, especially DNA methylation, is a potential way for humans to overcome cancer. Research on colorectal cancer shows that 5-Aza-2′-deoxycytidine (5-AzaDC) is a DNMT inhibitor, which can significantly reduce the number and activity of colorectal CSCs and can inhibit the progression of colorectal cancer (Li et al., 2018). Therefore, we envision whether 5-AzaDC is also an anti-cancer treatment approach for patients with HCC.

The role of immune cells in tumors has received more and more attention. A large number of studies have shown the important role of immune regulation in HCC, and immune-related anti-tumor drugs are also appearing repeatedly. Studies have shown that CSF1 receptor (CSF1R)-mediated signal transduction plays an irreplaceable role in the differentiation and survival of the mononuclear phagocyte system, especially macrophages (Stanley and Chitu, 2014). CSF1R belongs to the type III protein tyrosine kinase receptor family, and binding to CSF1 or the more recently identified ligand IL-34 can induce receptor homodimerization and subsequently activate receptor signal transduction (Achkova and Maher, 2016). Some studies have confirmed that CSF1R + macrophages are associated with poor survival of various tumor types (Zhang et al., 2012; Pedersen et al., 2014), so therapies targeting CSF1R-related signal transduction pathways such as CSF1R inhibitors have been proven effective against cancer (Cannarile et al., 2017). In our study, we confirmed the correlation between CSF1R (Rho = 0.396, *p* = 9.32e-16) and VCAN in HCC through DISIDB, which implies their positive correlation. Our research shows that low expression of VCAN can significantly improve the survival time of HCC patients, which implied that VCAN may be the downstream or upstream target of CSF1R in HCC, and is partly involved in its cancer-promoting effect. In addition, we have also unearthed many immunoinhibitors like CSF1R related to VCAN, such as CD96, PDCD1, CD271, etc., which are positively correlated with the expression of VCAN. In addition to immunoinhibitors, we also found that some immunostimulators such as CD28, CD86, CD27, lymphocytes such as macrophagesand Act-DC are all positively correlated with the expression of VCAN. It is suggested that VCAN plays an important role in immune regulation in HCC. Therefore, the combination of inhibitors against these immunological checkpoints and VCAN inhibitors may potentially enhance the anti-cancer effect in patients with HCC.

Our single-cell data show that VCAN is expressed in fibroblasts. The study has shown that VCAN mRNA specifically expressed in cancer-associated fibroblasts was further confirmed to be a prognostic factor in two additional independent datasets in 453 and 89 stages II/III patients (Chida et al., 2016). Additional studies have demonstrated using VCAN-negative QRsP11 fibrosarcoma cells that VCAN is an important molecule in functional ECM and maintenance of cancer-associated fibroblasts (Fanhchaksai et al., 2016). In addition to this, the high expression levels of CAF-related molecules including VCAN, periostin, and lumican in the stroma of ESCC were significantly associated with worse recurrence-free survival (RFS) and overall survival in ESCC patients (Yamauchi et al., 2021). In addition, TGF-β enhances ovarian cancer cell invasiveness by up-regulating VCAN in CAFs. VCAN expression is regulated in CAF by TGF-β receptor type II and SMAD signaling. Up-regulated VCAN promotes motility and invasion of ovarian cancer cells by activating the NF-κB signaling pathway and up-regulating the expression of CD44, matrix metalloproteinase 9, and hyaluronan-mediated motility receptors (Yeung et al., 2013). Both these studies and the results presented here support a role for VCAN in cancer-associated fibroblasts.

As mentioned in this article, the expression of VCAN is increased in many cancers, including HCC, which is significantly related to the poor prognosis of patients with HCC. At the same time, further analysis from Starbase showed that a variety of miRNA and circRNA related to VCAN expression, suggesting that these miRNAs and circRNAs may regulate VCAN and promote the progression of HCC. However, we have not carried out further experimental verification, which will be further improved in our follow-up research. Our study provides a new anti-hepatoma idea to find some DNA methyltransferase inhibitors and TF inhibitors that can effectively down-regulate the expression of VCAN. To sum up, VCAN has great potential to become a prognostic marker and therapeutic target for HCC.

## Conclusion

In conclusion, we systematically analyzed the expression profile and prognostic value of VCAN in HCC and predicted the possible biological functions and potential targeted therapeutic value of VCAN. Overall, our study provides systematic insights into the heterogeneous and complex roles of VCAN in HCC carcinogenesis.

## Data Availability

The original contributions presented in the study are included in the article/Supplementary Materials, further inquiries can be directed to the corresponding authors.
