# Supplementary material for: Comprehensive Analysis of VCAN Expression Profiles and Prognostic Values in HCC
Source: Front Genet. 2022 Jun 24;13:900306. doi: 10.3389/fgene.2022.900306 (PMC9263583; doi:10.3389/fgene.2022.900306)
Supplement: Supplementary file 1 [file DataSheet1.PDF]

Supplementary Table 1

TCGAportal	www.tcgaportal.org	To investigate the expression of VCAN in human tissues
Human protein **Atlas**	https://www.proteinatlas.org/	To detect VCAN's distribution and suHCCellular localization
UALCAN	http://ualcan.path.uab.edu/	To analyze the VCAN mRNA expression in different races, ages, molecular subtypes of HCC patients
KaplanMeier Plotter	http://kmplot.com/analysis/index.php?p=background	To analyzed the correlations between VCAN mRNA expression and RFS as well as OS
Cistrome DB Toolkit database	http://dbtoolkit.cistrome.org	Selected the TF of VCAN - FOXM1
MEXPRESS	https://mexpress.be/	To unearth the methylation details of VCAN as well as the relationship between VCAN mRNA expression and different clinical characteristics of HCC
GEPIA2	http://gepia2.cancer-pku.cn/#index	To assess the correlations between genes
TargetScanHuman	http://www.targetscan.org/vert_71/	To predict the miRNAs related to VCAN
starBase v3.0	http://starbase.sysu.edu.cn/index.php	To predict the miRNAs related to VCAN; To perform circRNAs prediction, survival analysis of miRNAs as well as correlation analysis between miRNAs and VCAN mRNA
Metascape	http://metascape.org/gp/index.html#/main/step1	To obtained the heatmap and network of enrichment terms related to VCAN
STRING	https://string-db.org/cgi/input.pl	To obtain the interaction network between VCAN and other important proteins
DISIDB	http://cis.hku.hk/TISIDB/index.php	To analyze the correlations between expressions of VCAN mRNA and 3

		kinds of immune factors
