# Supplementary figures and images for: Comprehensive Analysis of VCAN Expression Profiles and Prognostic Values in HCC
Source: Front Genet. 2022 Jun 24;13:900306. doi: 10.3389/fgene.2022.900306 (PMC9263583; doi:10.3389/fgene.2022.900306)

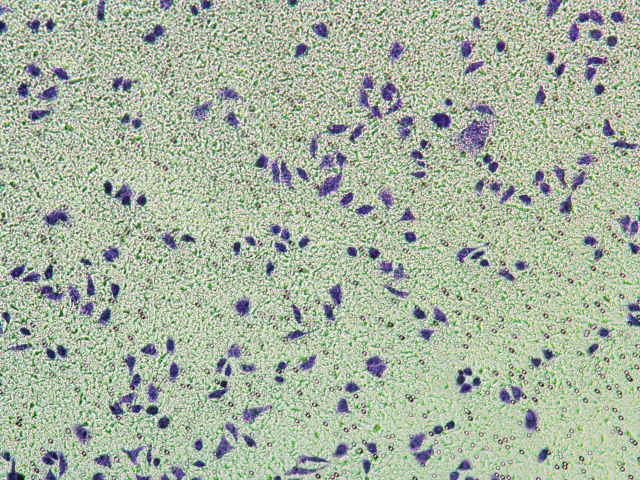

Supplement: Supplementary file 2 [file DataSheet2.ZIP › sgs vcan transwell/01.png]

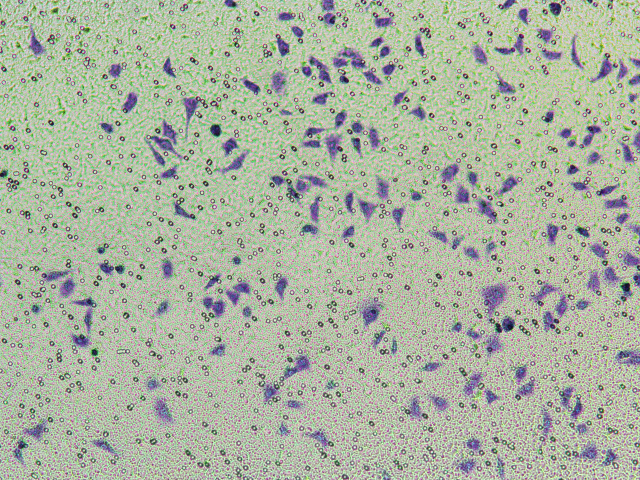

Supplement: Supplementary file 2 [file DataSheet2.ZIP › sgs vcan transwell/02.png]

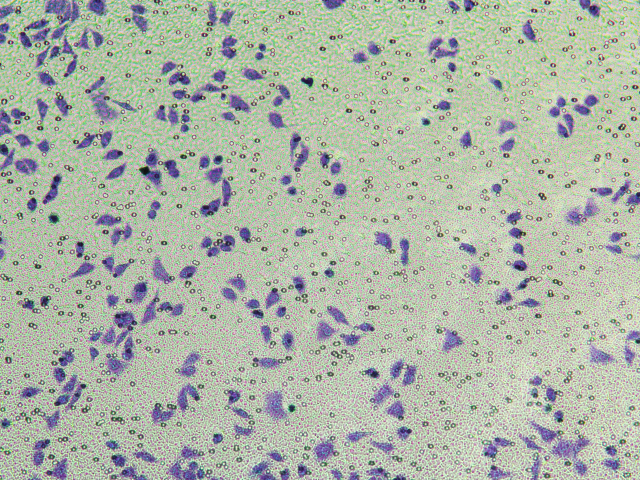

Supplement: Supplementary file 2 [file DataSheet2.ZIP › sgs vcan transwell/03.png]

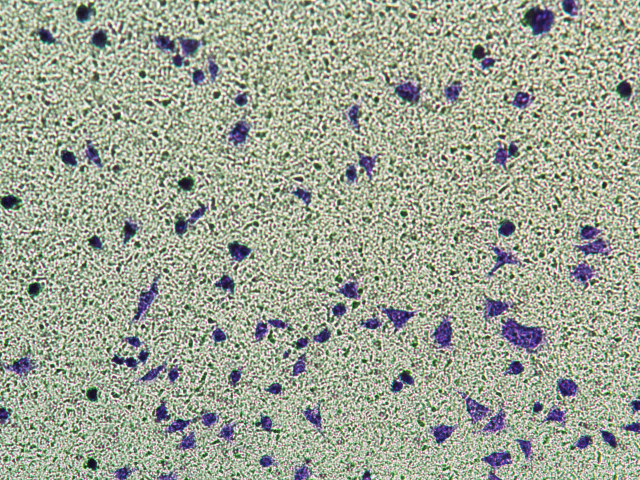

Supplement: Supplementary file 2 [file DataSheet2.ZIP › sgs vcan transwell/04.png]

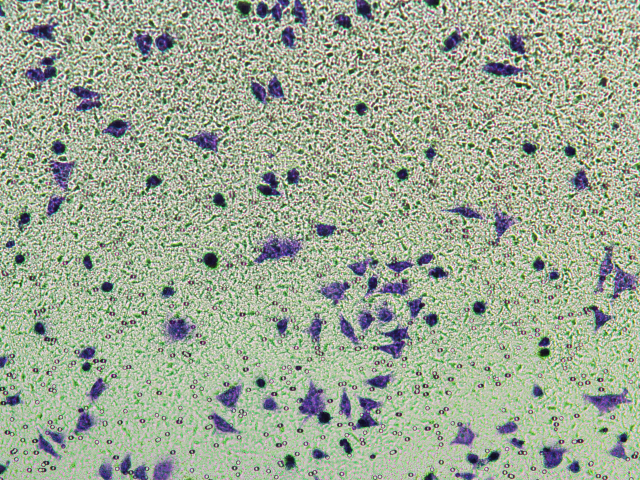

Supplement: Supplementary file 2 [file DataSheet2.ZIP › sgs vcan transwell/05.png]

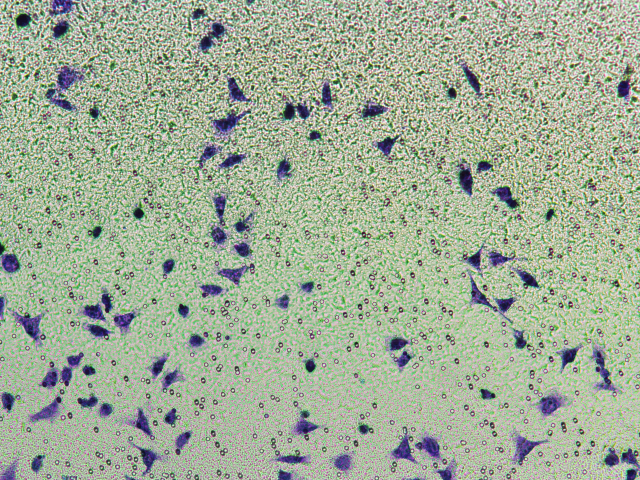

Supplement: Supplementary file 2 [file DataSheet2.ZIP › sgs vcan transwell/06.png]

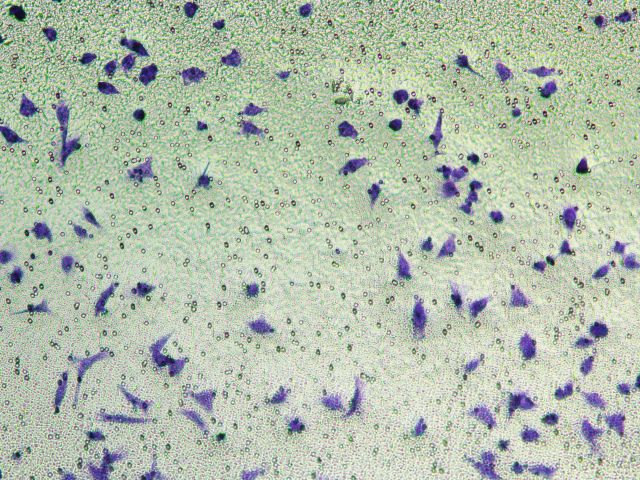

Supplement: Supplementary file 2 [file DataSheet2.ZIP › sgs vcan transwell/07.png]

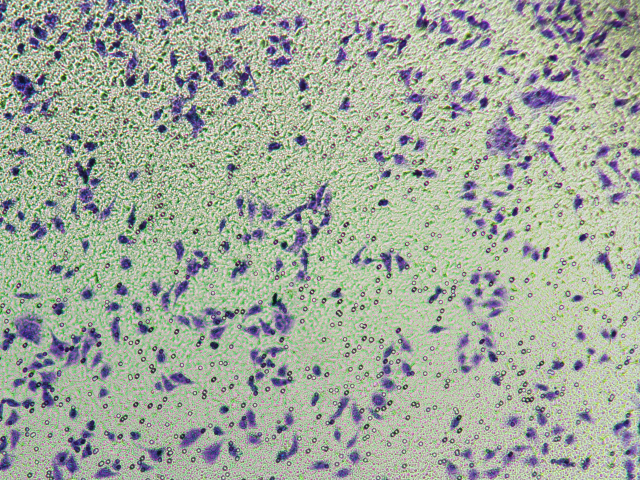

Supplement: Supplementary file 2 [file DataSheet2.ZIP › sgs vcan transwell/08.png]

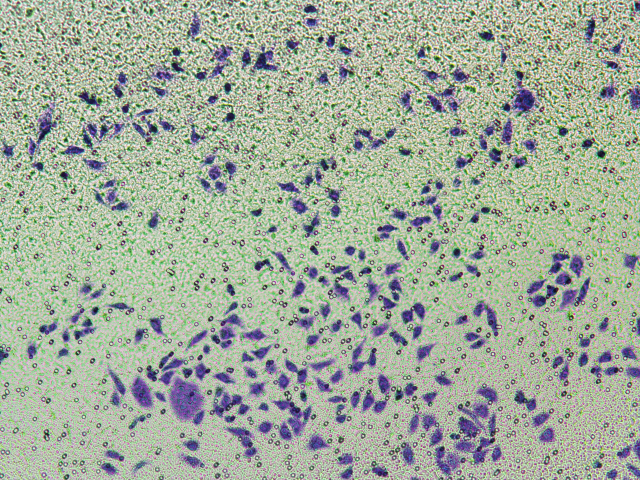

Supplement: Supplementary file 2 [file DataSheet2.ZIP › sgs vcan transwell/09.png]

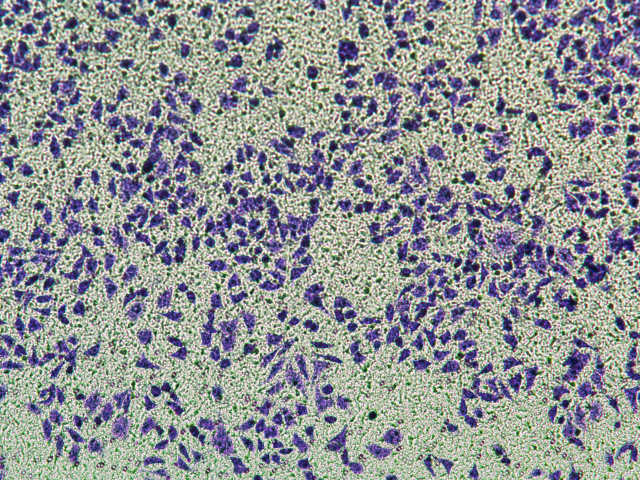

Supplement: Supplementary file 2 [file DataSheet2.ZIP › sgs vcan transwell/10.png]

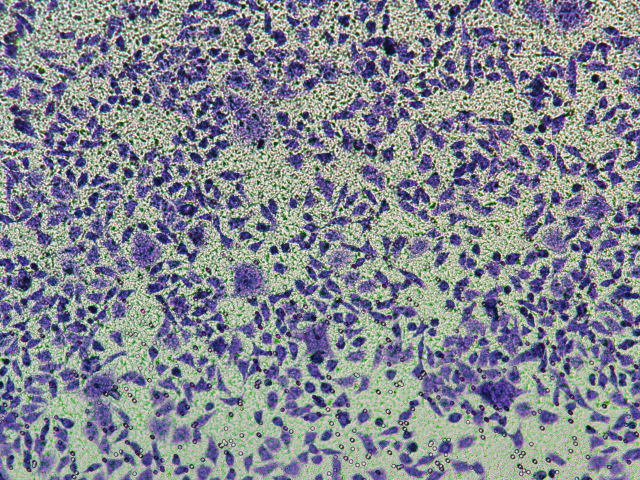

Supplement: Supplementary file 2 [file DataSheet2.ZIP › sgs vcan transwell/11.png]

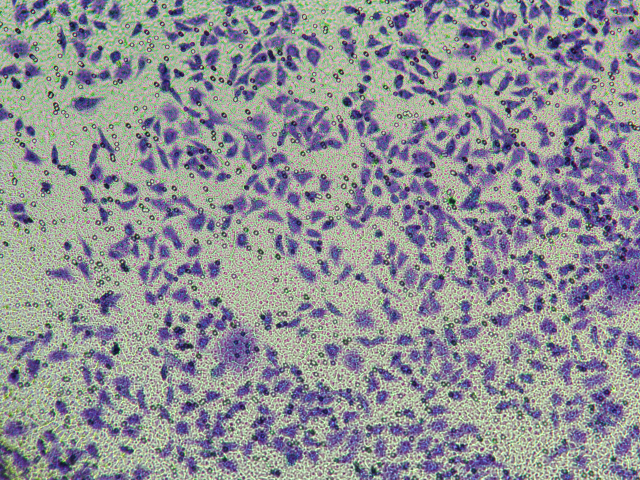

Supplement: Supplementary file 2 [file DataSheet2.ZIP › sgs vcan transwell/12.png]

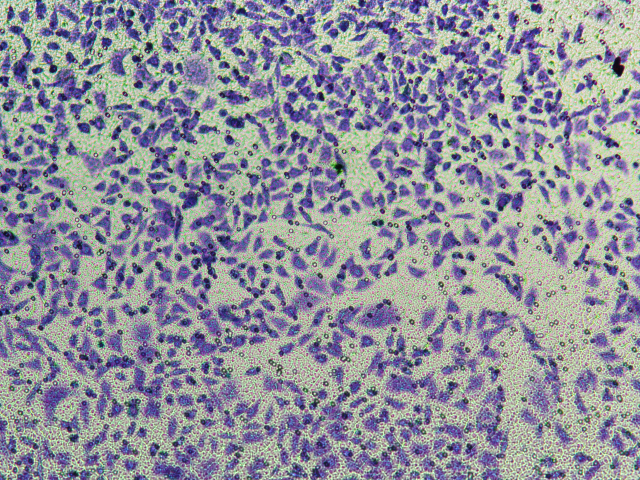

Supplement: Supplementary file 2 [file DataSheet2.ZIP › sgs vcan transwell/13.png]

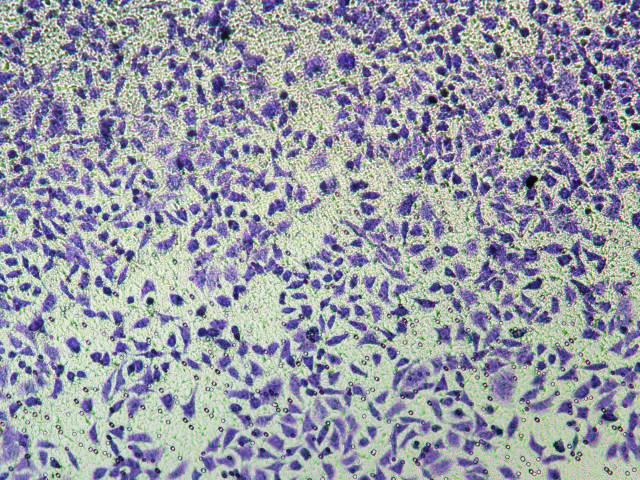

Supplement: Supplementary file 2 [file DataSheet2.ZIP › sgs vcan transwell/14.png]

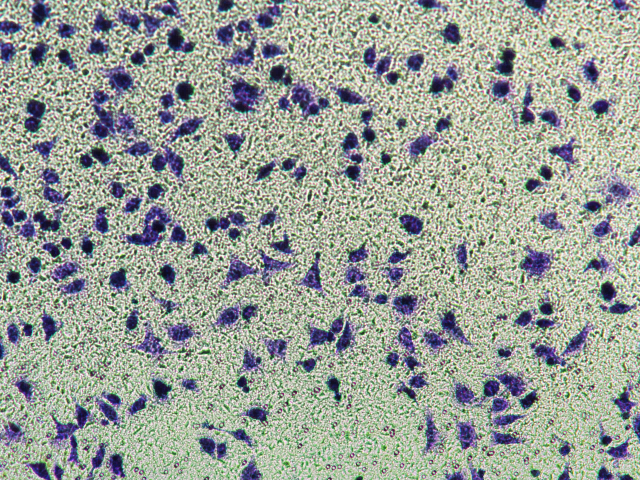

Supplement: Supplementary file 2 [file DataSheet2.ZIP › sgs vcan transwell/15.png]

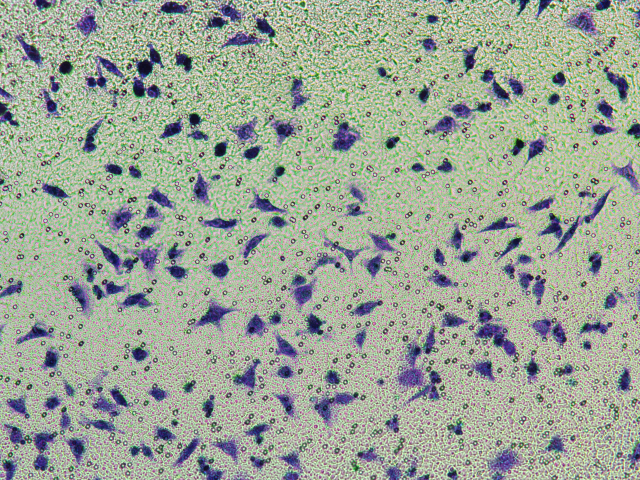

Supplement: Supplementary file 2 [file DataSheet2.ZIP › sgs vcan transwell/16.png]

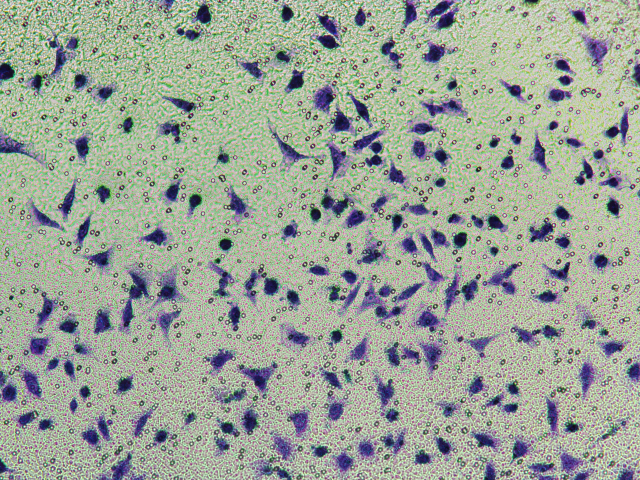

Supplement: Supplementary file 2 [file DataSheet2.ZIP › sgs vcan transwell/17.png]

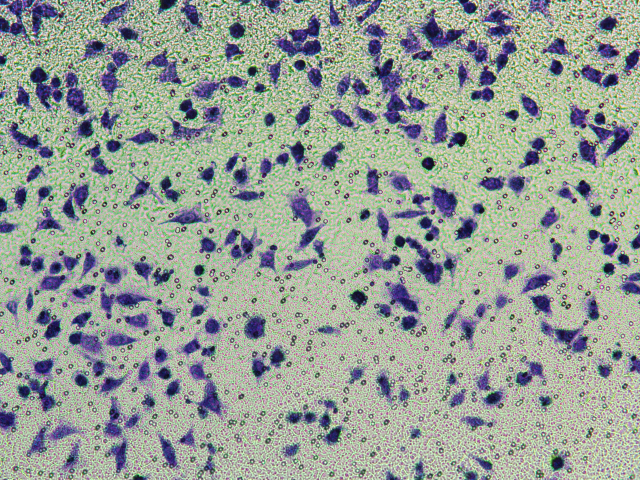

Supplement: Supplementary file 2 [file DataSheet2.ZIP › sgs vcan transwell/18.png]

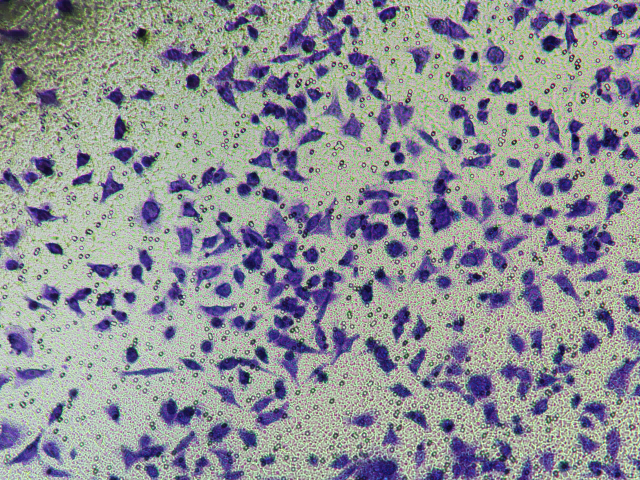

Supplement: Supplementary file 2 [file DataSheet2.ZIP › sgs vcan transwell/19.png]

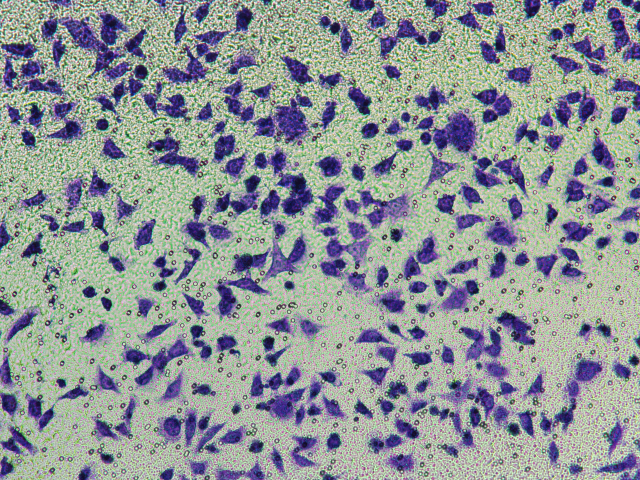

Supplement: Supplementary file 2 [file DataSheet2.ZIP › sgs vcan transwell/20.png]

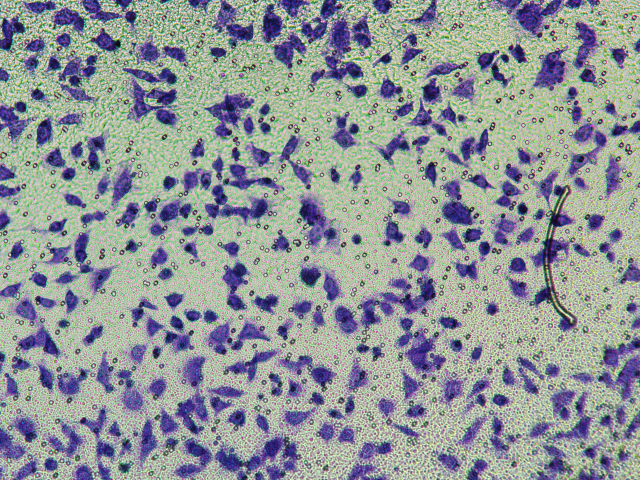

Supplement: Supplementary file 2 [file DataSheet2.ZIP › sgs vcan transwell/21.png]

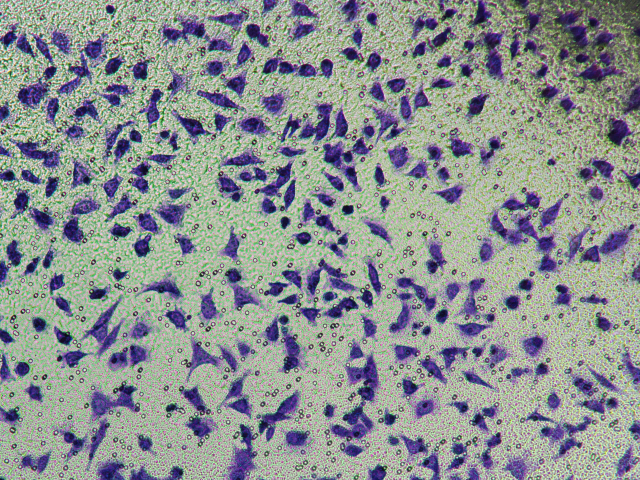

Supplement: Supplementary file 2 [file DataSheet2.ZIP › sgs vcan transwell/22.png]
